# Phage Display in Cancer Research: Special Issue Editorial

**DOI:** 10.3390/v16060968

**Published:** 2024-06-17

**Authors:** Valery A. Petrenko

**Affiliations:** Department of Pathobiology, College of Veterinary Medicine, Auburn University, Auburn, AL 36849, USA; petreva@auburn.edu

## 1. Introduction

**Figure 1:**
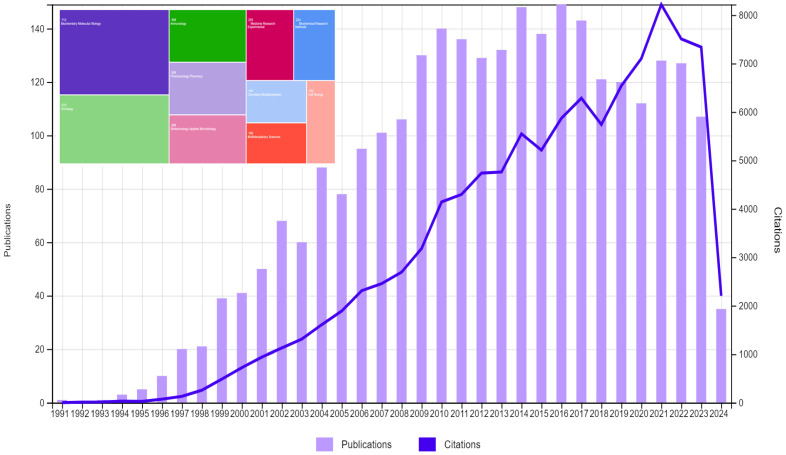
Topic phage display + cancer. Times cited and publications over time. Calculated by using Clariwait and Web of Science^TM^.

Soon after its birth in 1985, following a short lag period [1,2], the phage display technology enjoyed a tremendous exponential growth, demonstrating its competence and performance in solving numerous challenging problems in material science, bioengineering, and medicine, including cancer research [3,4,5,6,7,8,9,10,11,12,13] (Figure 1). This Special Issue aims to illustrate the promise of phage display as a universal research tool in cancer research. We chose manuscripts that bring the fundamental principles of phage display to a new level in the development of cancer diagnostics and immunotherapeutics.

## 2. Discussion

This Special Issue contains three reviews and two original studies that contribute to the overall knowledge of phage display applications in cancer research.

The comprehensive review **“Improving Pharmacokinetics of Peptides Using Phage Display”**, prepared by Mallika Asar et al. [14], shows how phage display technology can be used to enhance target specificity, which refers to the biodistribution and clearance of peptides that are used in the targeting of disease-relevant biomarkers.

Another comprehensive review chosen for this Special Issue, **“Progress on Phage Display Technology: Tailoring Antibodies for Cancer Immunotherapy”**, presented by Renato Kaylan Alves França and coauthors], unveils the remarkable progress in this field and the possibilities of using clever strategies for phage selection and tailoring the refinement of antibodies aimed at increasingly specific targets.

Finally, the review article **“Phage Display’s Prospects for Early Diagnosis of Prostate Cancer”**, written by Valery A. Petrenko, describes the success story of how the search for the “Holy Grail” of cancer researchers and bioengineers led to the design of phage-based molecular sensing probes that allow for the diagnosis, prognosis, and monitoring of cancer diseases via their interaction with cell-secreted and cell-associated cancer biomarkers. This review describes the role of the molecular evolution and phage display paradigm in revolutionizing the methods for the early diagnosis and monitoring of prostate cancer.

Two interesting research papers present original technical aspects of phage display technology, leading to the enhancement of its performance in the engineering of cancer medicines and diagnostics.

In the paper **“Combining Cellular Immunization and Phage Display Screening Results in Novel, FcγRI-Specific Antibodies”**, by Steffen Krohn et al. [15], the authors describe how hybridoma technology was replaced with phage display of a single-chain fragment variable (scFv) antibody library that was generated from mice who were immunized with FcγRI-positive cells and screened with a cellular panning approach, assisted by next-generation sequencing (NGS). Seven new FcγRI-specific antibody sequences were selected in this methodology, which were produced as Fc-silent antibodies showing FcγRI-restricted specificity.

The paper **“Phage vs. Phage: Direct Selections of Sandwich Binding Pairs”**, by Emily C. Sanders et al. [16], presents an advanced phage display selection method that allows for the development of peptides and Fabs ligands for sandwich immunoassays. The approach was exemplified by the preparation of two sandwich pairs, one peptide–peptide and one Fab–peptide sandwich, for cancer and Parkinson’s disease biomarkers. The authors assume that the results of their study can be applied for the development of binding partners for a wide range of clinical biomarker assays.
